# Optimization of Haskap Extract and Tannic Acid Combined with Mild Heat Treatment: A Predictive Study on the Inhibition of *Cronobacter sakazakii*

**DOI:** 10.3390/foods14040562

**Published:** 2025-02-08

**Authors:** Gökçe Polat Yemiş, Oktay Yemiş, Aysun Öztürk

**Affiliations:** 1 Department of Food Engineering, Faculty of Engineering, Sakarya University, Sakarya 54187, Turkey; oktayyemis@sakarya.edu.tr; 2 Sakarya University Research, Development, and Application Center (SARGEM), Sakarya University, Sakarya 54050, Turkey; 3 Department of Food Technology, Atatürk Horticultural Central Research Institute, Yalova 77102, Turkey; aysun.ozturk@tarim.gov.tr

**Keywords:** *Cronobacter sakazakii*, powdered infant formula, haskap polyphenol extract, antibacterial activity, optimization

## Abstract

*Cronobacter sakazakii* is an opportunistic food-borne pathogen that causes severe infections with high morbidity and mortality rates in neonates, the elderly, and immunocompromised individuals. The plant extracts containing natural antibacterial compounds are currently under consideration as alternatives to synthetic artificial preservatives for the control of *C. sakazakii.* There has been increasing interest in using plant-derived antimicrobials in combination with mild heat to control pathogens in preservative-free foods. In this study, the individual and combined effects of four independent variables, i.e., polyphenol-rich haskap extract (HE) concentration (2–10%), tannic acid (TA) concentration (0.1–0.5), temperature (35–55 °C), and time (1–5 min), on *C. sakazakii* inactivation were investigated by response surface methodology (RSM) with a five-level four factor central composite design (CCD) and an optimal combination for maximum inhibition was determined. The statistic metrics of *R^2^*, *R^2^_adjusted_*, *R^2^_predicted_*, coefficient of variation (CV), Predicted Residual Error Sum of Squares (PRESSs), adequate precision, and lack-of-fit were used to reveal the prediction performance. The results revealed that all the independent variables, except time, influenced *C. sakazakii* inactivation. Among the independent variables, the temperature was the most effective variable (*p* < 0.0001) as regards inactivation. The synergistic effects of HE with TA and temperature were observed. Many possible optimum conditions of mild heat treatment that maximized the inhibition of *C. sakazakii* were obtained. The findings indicated that two distinct combinations were identified as the most effective inhibition of *C. sakazakii*: high concentration at low temperature and high temperature at low concentration. It can be concluded that haskap polyphenol extract, alone or in combination with tannic acid, has the potential to be used as a natural preservative to reduce the risk of *C. sakazakii*.

## 1. Introduction

*Cronobacter sakazakii* is an emerging foodborne pathogen, causing life-threatening diseases like meningitis, necrotizing enterocolites, and bacteremia with 40–80% estimated case-fatality rates, and severe neurological sequelae upon recovery in newborns and premature infants [1,2,3]. The International Commission on Microbiological Specifications for Foods has identified *C. sakazakii* as a “severe hazard for restricted populations, life-threatening or substantial chronic sequelae of long duration” [4]. *C. sakazakii* has been isolated from a wide range of foods and food components, as well as from various environmental and clinical sources, including hospitals, homes and factories. Powdered infant formula (PIF) has been reported as the main source of neonatal infection with *C. sakazakii* in the literature [5,6,7]. The bacterium shows a remarkable resistance to desiccation heat, dry and acid stress growth conditions compared to other members of the family *Enterobacteriaceae*, which may contribute to their long survival in PIF and on surfaces in the production environment [8,9,10,11]. *C. sakazakii* contamination can occur at any stage of PIF production, particularly in raw ingredients, as a consequence of environmental contamination during processing, insufficient washing procedures and the preparation of PIF intended for infant consumption. Moreover, several studies have demonstrated that certain strains of *C. sakazakii* have the ability to form biofilms on a range of materials, including glass, stainless steel, polyvinyl chloride, polycarbonate, silicone, and enteral feeding tubes. This biofilm formation represents a significant concern for the PIF industry as it suggests that biofilms on raw materials or surfaces in contact with PIF may serve as potential sources of contamination with *C. sakazakii* [12,13].

Recently, there has been interest in the potential use of plant-derived antimicrobials in combination with mild heat to control pathogens in preservative-free foods. Natural antibacterial compounds derived from plants are currently under consideration as alternatives to synthetic artificial preservative for the control of *C. sakazakii* [14,15]. The haskap berry (*Lonicera caerulea* L.), commonly known as the honeyberry or blue honeysuckle, is native to the cool temperate regions of the Northern Hemisphere. It has recently been cultivated as a berry crop in North America [16]. The haskap berry is a rich source of nutrients and bioactive compounds, including polyphenols, flavonoids, anthocyanins, minerals, and secondary metabolites with bioactive properties, which have been the subject of considerable research interest due to their potential health-promoting properties [17,18]. The principal classes of phenolics present in haskap are flavanols (quercetin glycosides), flavan-3-ols (catechin and proanthocyanidins), anthocyanins (cyanidin glucoside) and phenolic acids (salicylic acid, coumaric acid and gentistic acid) [19,20,21]. A number of in vitro studies have demonstrated that the haskap berry, which is rich in polyphenolic compounds, exhibits antibacterial activity against both Gram-negative and Gram-positive bacteria [20,22,23,24,25]. Tannic acid, a water-soluble natural polyphenol compound, has been approved by the Food and Drug Administration (FDA) as a safe food additive. It has been demonstrated to exert bacteriostatic and bactericidal effects against a diverse range of microorganisms, including foodborne bacterial pathogens [26,27,28,29]. 

The mathematical modeling of the behavior of microorganisms under different environmental conditions (i.e., pH, temperature, natural antimicrobial agents) is an effective and economical tool for the prediction of food safety, as is the prediction of microorganism inactivation. The conventional “one-factor-at-a-time” strategy is a time-intensive method which does not consider the interaction between independent variables. In contrast, response surface methodology (RSM) is an empirical modeling technique that can evaluate the many independent variables and their interactions affecting the response with fewer experiments [30]. RSM has been successfully used for studying the synergetic effects of combined treatments for the growth and behavior of foodborne pathogens by many researchers [31,32,33,34]. In our previous study, the antibacterial properties of haskap berry extract and tannic acid were investigated against *Cronobacter* species, and their potential application as antibacterial agents in powdered infant formula was determined. Our findings revealed that both Haskap extract and tannic acid had bacteriostatic and bactericidal activity against all tested *Cronobacter* species. Haskap extract and tannic acid supplementation of powdered infant formula reduced the thermal resistance of *C. sakazakii* during heating at 55 °C and inactivated the species in the reconstituted product during storage [20]. The objectives of this study were to (1) investigate the individual and interactive effects of haskap berry extract concentration, tannic acid concentration, temperature and time on the inactivation of *C. sakazakii*, (2) determine the possible optimum conditions for the maximum inhibition using response surface methodology.

## 2. Materials and Methods

### 2.1. Haskap Berry Fruit and Extraction

In this study, the Indigo Gem variety of haskap fruit (*Lonicera caerulea* L.) harvested from a berry farm (Summerland Haskap, Summerland, BC, Canada) in the Okanagan Valley was used. The fresh berries after frozen at −20 °C were lyophilized by a freeze dryer (The Virtis Company, Gardiner, NY, USA) for 3 days at 150 mTorr.

Haskap extract (HE) was obtained by ultrasound-assisted extraction, as detailed in the previous study of Yemiş et al. [20]. Briefly, 2 g of powdered-form berry samples, which were freeze-dried in 48 mL of a solution containing 80% MeOH (0.01% HCl, *v*/*v*), was ultrasonicated by using an ultrasonic bath (Bandelin, Sonorex, RK 156, Germany) for 30 min at room temperature. The supernatants centrifuged at 8000 rpm for 10 min were filtered through Whatman No. 1 filter and then concentrated by removing methanol at 40 °C using an evaporator (Büchi, R114, Flawil, Switzerleand). The aqueous haskap extracts, which were volumeted with acidified water (0.01% HCl, *v*/*v*) to a final volume of 50 mL, were stored in a freezer (−40 °C) until optimization treatments. 

### 2.2. Bacterial Strain and Cultural Conditions

*C. sakazakii* (ATCC 29544, American Type Culture Collection, Manassas, VA, USA) was used for the bacterial inhibition treatments. The stock culture of *C. sakazakii* was preserved in tryptic soy broth (TSB; Merck, Darmstadt, Germany) supplemented with 20% glycerol at −20 °C. The stock culture was activated for 24 h at 37 °C in TSB, which contained 5 g/liter yeast extract (TSBYE).

### 2.3. Experimental Design and Model Development

Response surface methodology based on the central composite design was performed to examine the effects of each factor and to predict the optimal conditions for the maximum reduction in *C. sakazakii*. Haskap polyphenolic extract concentration (*X_1_*), tannic acid concentration (*X_2_*), temperature (*X_3_*), and time (*X_4_*) were independent variables (Table 1). The response was a logarithmic reduction (log cfu/mL) in *C. sakazakii*. The ranges of the independent variables were chosen based on results from a preliminary study. The experimental design contained 28 treatments, including 4 replicates at the center points. Each treatment consisting of experimental combinations was carried out in triplicate, and the mean reduction was presented as the response. 

The data were analyzed using the statistical software package of Design Expert 7.00 (Stat-Ease Inc., Minneapolis, MN, USA) to produce a regression model and determine the optimal conditions. Analysis of variance (ANOVA) was used to estimate the importance of the suggested regression model and individual model coefficients. The following quadratic polynomial equation was used.

*Y* = *k_0_ + k_1 × 1_* + *k_2 × 2_* + *k_3_X_3_* + *k_4_X_4_* + *k_5_X_1_^2^* + *k_6_X_2_^2^* + *k_7_X_3_^2^* + *k_8_X_4_^2^* + *k_9_X_1_X_2_* + *k_10_X_1_X_3_* + *k_11_X_1_X_4_* + *k_12_X_2_X_3_* + *k_13_X_2_X_4_* + *k_14_X_3_X_4_*

where Y is the predicted response (logarithmic reduction); *k_0_* is the interception coefficient; *k_1_*, *k_2_*, *k_3_* and *k_4_* are coefficients of the independent variables; *k_5_*, *k_6_*, *k_7_* and *k_8_* are coefficients of quadratic effects; and *k_9_*, *k_10_*, *k_11_*, *k_12_*, *k_13_* and *k_14_* are interaction coefficients. The 3D surface response plots of equations, which represent the relationships between the response and the independent variables, were created. We accepted that *p*-values of less than 0.05 were statistically significant. The ANOVA outputs, including *R^2^*, *R^2^_adjusted_*, *R^2^_predicted_*, coefficient of variation (CV), Predicted Residual Error Sum of Squares (PRESSs), adequate precision, lack-of-fit, *p*, and Fisher’s test values (*F*-value), were used to evaluate the fit of the models. All experiments were carried out in triplicate. The data were displayed as mean values ± standard deviations.

### 2.4. Bactericidal Activity

Stock solutions of haskap polyphenolic extract and tannic acid in a 0.85% NaCl solution adjusted to pH 5.0 were sterilized by filtration through 0.45 µm cellulose acetate membrane filters (Isolab, Eschau, Germany). Sterile culture tubes containing the required amounts of stock solution were supplemented with the stock solution to achieve final concentrations of 2, 4, 6, 8, and 10% haskap polyphenolic extract and 0.1, 0.2, 0.3, 0.4, and 0.5% tannic acid. The tubes were preheated at the designated temperature (35, 40, 45, 50, and 55 °C) in a temperature-controlled shaking water bath (Precision Scientific Inc., Chicago, IL, USA) before the experiment. *C. sakazakii* culture was added to each test tube to achieve an initial cell density of approximately 7 log cfu/mL. The samples were withdrawn from the test tube at designated times (1, 2, 3, 4, and 5 min) and immediately put into an ice-water bath and plated. The surviving cell populations in the samples were determined by the spread plate method on TSYE and incubated at 37 °C for 24 h. The recovery of cells below the detection limit was achieved through the enrichment of a 1 mL sample in 10 mL of fresh TSBYE at 37 °C for 24 h.

## 3. Results and Discussion

### 3.1. Development of Mathematical Equations for the Prediction of C. sakazakii Inhibition

The experimental design of the central composite as four factors–five levels and the experimental inhibition values under these conditions are given in Table 2. The obtained data were analyzed by the Design-Expert package program, and the significance of the possible regression models and the coefficients in the model (significance test) was revealed by analysis of variance (ANOVA). Four models, including linear, factorial, quadratic, and cubic, were compared to determine a suitable model. The adequacy of linear, factorial, quadratic, and cubic models was evaluated on the basis of *F*, *p*, lack-of-fit, *R^2^*, *R^2^_adjusted_* and *R^2^_predicted_* values (Table 3). The linear model had the lowest *p*-value (<0.0001) which means that the model is highly significant. However, the other parameters, including lack-of-fit, *R^2^*, *R^2^_adjusted_* and *R^2^_predicted_* for the adequacy and fitness of the linear model, were not satisfactory compared to the quadratic model. The quadratic model had a *p*-value of 0.008, *R^2^* of 0.9741, *R^2^_adjusted_* of 0.9463, and *R^2^_predicted_* of 0.8551, which are statistical metrics for evaluation of the adequacy and fitness of the model. Compared to the linear model, these critical statistic values of the quadratic model were higher, which means that the quadratic model represents a more reliable relationship between variables and response. The ‘fit summary’ report produced by Design-Expert indicated that the quadratic model was statistically the most suitable model, considering all these obtained criteria for the reduction. 

ANOVA analysis was performed on this proposed quadratic model, and the regression equation expressing the relationship between the process variables and each response was created. For this purpose, the “linear” effect terms of each variable, and the “quadratic” and “interaction” terms were examined. The regression coefficients for the quadratic model proposed by the design and their importance levels are given in Table 4. The proposed quadratic model was found to be significant (*p* < 0.0001). By using these coefficients, the following mathematical Equation (1) was reached:

Y (log reduction) = 47.87 − 2.80 *X_1_* + 9.1 *X_2_* − 2.07 *X_3_* − 1.88 *X_4_* + 1.40 *X_1_X_2_* + 0.04 *X_1_X_3_* + 0.04 *X_1_X_4_* − 0.32 *X_2_X_3_* − *2*.64 *X_2_X_4_* + 0.03 *X_3_X_4_* + 0.07 *X_1_^2^* + 15.96 *X_2_^2^* + 0.03 *X_3_^2^* + 0.23 *X_4_^2^* (1)

As can be seen in Table 4, the linear terms of HE concentration (*X_1_*), TA concentration (*X_2_*), temperature (*X_3_*), the interaction term of HE concentration*TA concentration (*X_1_X_2_*), HE concentration*temperature (*X_1_X_3_*), the quadratic term of HE concentration (*X_1_^2^*), temperature (*X_3_^2^*), and time (*X_4_^2^*) had significant effects (*p*-values < 0.05) on the inactivation of *C. sakazakii*. However, the linear term of time (*X_4_),* the interaction term of HE concentration*time (*X_1_X_4_*), TA concentration*temperature (*X_2_X_3_*), TA concentration*time (*X_2_X_4_*), temperature*time (*X_3_X_4_*), *and* the quadratic term of TA concentration (*X_2_^2^*) were statistically insignificant at p>0.05. In order to simplify the model, the insignificant terms (*X_1_X_4_*, *X_2_X_3_*, *X_2_X_4_*, *X_3_X_4_*, *X_2_^2^),* in the quadratic model were removed by performing the process of backward elimination (α = 0.05). The coefficients for the reduced quadratic model, which contains only the significant terms, and their significance levels are given in Table 5. By using these coefficients, the mathematical equation (Equation 2) below was produced. Although the linear term of time (*X_4_*) was insignificant (*p* > 0.05), the quadratic term (*X_4_^2^*) was significant in the proposed model. So, the linear term of time (*X_1_*) was hierarchically added to the quadratic equation.

Y (log reduction) = 44.95 *−* 2.54 *X_1_* − 3.54 *X_2_ −* 1.94 *X_3_ −* 0.95 *X_4_ +* 1.40 *X_1_X_2_ +* 0.04 *X_1_X_3_ +* 0.06 *X_1_^2^ +* 0.02 *X_3_^2^ +* 0.19 *X_4_^2^* (2)

**Figure 1:**
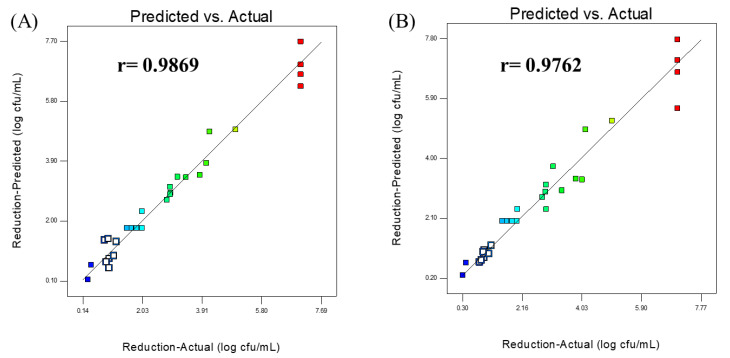
Scatter diagram of the predicted versus the actual values for the quadratic (**A**) and the reduced quadratic (**B**) model. r: the correlation coefficient between the predicted and the actual values of the models.

The *p*-value of the reduced quadratic model was lower than 0.0001, similar to the aforementioned p-value of the quadratic model, which was significant. The statistical metrics of *R^2^*, *R^2^_adjusted_*, *R^2^_predicted_*, coefficient of variation (CV), Predicted Residual Error Sum of Squares (PRESSs), adequate precision, and lack-of-fit were used to evaluate the adequacy and fitness of the quadratic and reduced quadratic model. The descriptive statistical metrics of both the quadratic and the reduced quadratic model are given in Table 4. The determination coefficient (*R^2^*), *R^2^_adjusted_*, and *R^2^_predicted_* values were 0.9741, 0.9463, and 0.8551, respectively, for the quadratic model, whereas the values of *R^2^*, *R^2^_adjusted_*, and *R^2^_predicted_* were 0.9531, 0.9297, and 0.8622, respectively, for the reduced quadratic model. It is desired that the *R^2^*, *R^2^_adjusted_*, and *R^2^_predicted_* values should be high for a good prediction. The determination coefficient (*R^2^*) of 0.9741 means that the equation from the quadratic model could explain 97.41% of the variations and only 2.29% of the total variations could not be explained by the quadratic model. The difference between *R^2^_adjusted_* (0.9463) and *R^2^_predicted_* (0.8551) being of less than 0.2 implies that *R^2^_adjusted_* is in reasonable agreement with *R^2^_predicted_*. The values of *R^2^* and *R^2^_adjusted_* of the quadratic model were found to be higher than those of the reduced quadratic model, except the *R^2^_predicted_* value. These obtained values indicate that there is a high correlation between the experimental values and the predicted values. To support these findings, the predicted values calculated from the equations based on the quadratic and reduced quadratic model were compared to the experimental values (Table 2). The correlation coefficient (*r*) between the actual and predicted values was 0.9869 and 0.9762 for the quadratic and the reduced quadratic models, respectively. Figure 1 shows the plots formed by the actual values versus the predicted values produced from the quadratic and reduced quadratic models. The plots, which indicate almost all data are dispersed around the regression line, supported the high values of the calculated correlation coefficients for the quadratic and reduced quadratic models. The quadratic model had a higher correlation than the reduced quadratic model. This also shows that the quadratic model has a high predictive capability.

The adequate precision value representing the signal-to-noise should be at least 4 for a significant model [35]. In our study, the ratio was 20.97 and 22.22 for the quadratic and reduced quadratic models, respectively, which was much higher than the desired value of adequate precision. The predicted residual error sum of squares (PRESSs) statistic is a metric of the fitness of each data point in the design, and a smaller PRESSs value indicates a better predictability of the developed model [36]. The PRESSs values for the quadratic and reduced quadratic models were 17.60 and 16.74, respectively. Lack-of-fit is one of the key tests to evaluate the adequacy of the proposed models, and a *p*-value of more than 0.05 shows that the lack-of-fit of the models was insignificant, meaning data fit the model well. In our study, the lack-of-fit value was 0.0650 and 0.0484 for the quadratic and reduced quadratic models, respectively. Since the lack-of-fit value of the reduced quadratic model is lower than 0.05, it is concluded that this model cannot be used in prediction. The simplification by the backward elimination of the proposed model did not improve the predictive capability. Therefore, the quadratic model was considered to determine the individual and synergetic effects of independent variables and the prediction of optimum conditions for maximizing the inhibition of *C. sakazakii*. These results revealed that Equation (1), reflecting the quadratic model, provides a more accurate estimation for the inhibition of *C. sakazakii*.

### 3.2. Effect of Independent Variables on the Inhibition of C. sakazakii

**Figure 2:**
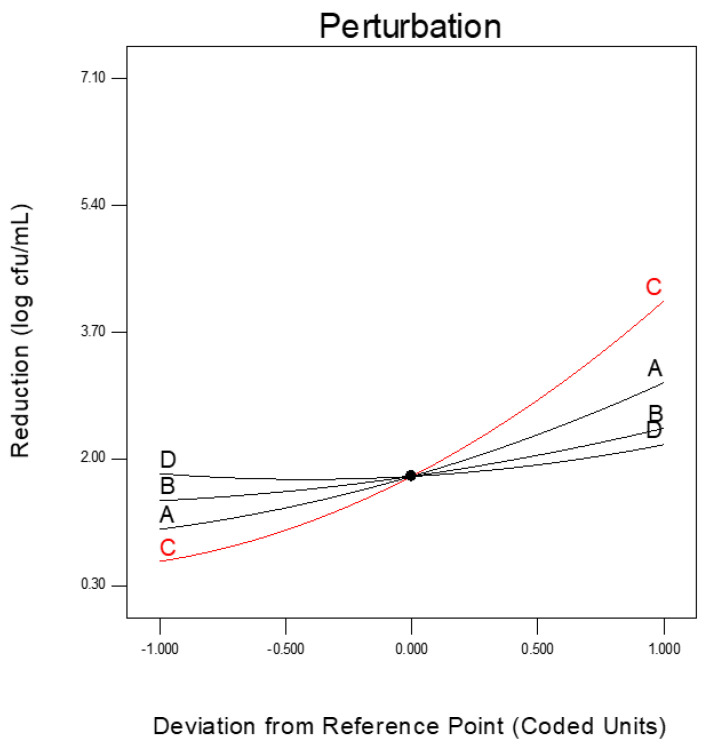
Perturbation plot showing the individual effects of HE concentration (A), TA concentration (B), temperature (C), and time (D) on the reduction in *C. sakazakii*.

In this study, four factors, including HE concentration (2–10%), TA concentration (0.1–0.5%), temperature (35–55 °C), and time (1–5 min), were considered independent variables, and their effects on the inhibition of *C. sakazakii* were evaluated. As can be seen from Table 2, the reduction values varied in the range of 0.31-7.04 log cfu/mL. The lowest reduction of 0.31 log cfu/mL was obtained with the first treatment, which consisted of an HE concentration of 4%, TA concentration of 0.2%, temperature of 40 °C, and time of 2 min. The highest reduction (7.04 log cfu/mL) was observed at with 8th, 14th, 16th, and 22nd treatments under different conditions. The treatments that provided the highest inhibition had combinations of high temperature with HE concentration. The analysis of variance (ANOVA) table (Table 4) presents the order of significance for each coefficient based on the probability values. We observed that among the independent variables, HE concentration (*X_1_*) and temperature (*X_3_*) were more effective factors than TA concentration on inactivation (*p* < 0.0001). On the other hand, it was determined that time (*X_4_*) had no effect (*p* > 0.05) on inactivation in the range of 1–5 min (Table 4). A similar result was reported by Kim and Rhee [37], who worked on the inactivation of *E. coli* O157:H7 of the combined treatment of citric and caprylic acid by using the surface response method. They determined that the treatment time had no influence on the reduction in *E. coli* O157:H7. Figure 2 shows the perturbation plot, which compares the effect of all independent variables at the center point. Sharp curvatures in the perturbation plot show a high sensitivity of the reduction to the related independent variables. As shown in the perturbation plot, temperature showed the maximum influence on the reduction in *C. sakazakii*, followed by HE concentration and TA concentration. There was a negligible effect of treatment time on the reduction.

**Figure 3:**
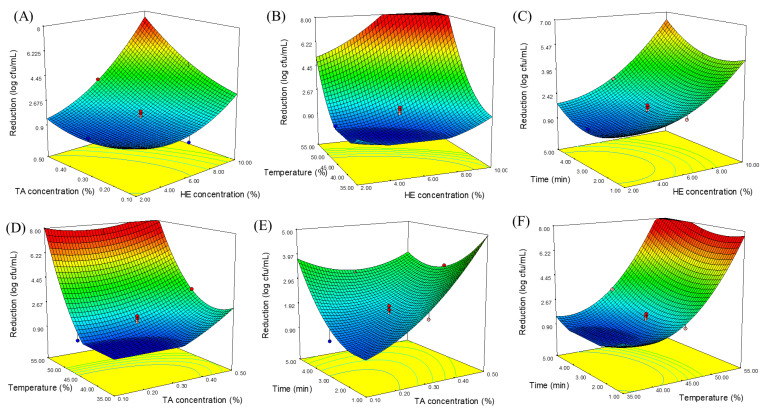
Response surface plot showing the effect of HE concentration, TA concentration, temperature and time on the inhibition of *Cronobacter sakazakii.* (**A**) Fixed at a temperature of 45 °C and time of 3 min, (**B**) fixed at a TA concentration of 0.3% and time of 3 min, (**C**) fixed at a TA concentration of 0.3% and temperature of 45 °C, (**D**) fixed at an HE concentration of 6% and time of 3 min, (**E**) fixed at an HE concentration of 6% and temperature of 45 °C, (**F**) fixed at an HE concentration of 6% and TA concentration of 0.3%.

The interactions HE concentration*TA concentration and HE concentration*temperature were significant at *p* < 0.05 (*p* = 0.0404) and *p* < 0.01 (*p* = 0.0043), respectively, whereas there were no interactions (*p* > 0.05, Table 4) between other independent variables (HE concentration*time, TA concentration*temperature, TA concentration*time, and temperature*time). Figure 3A shows the synergistic effect of the combined treatment of HE and TA. In our previous study [20], we revealed that the tannic acid and haskap extract prepared from cultivar Indigo Gem had bactericidal and bacteriostatic effects against *Cronobacter* spp. and determined that there is a synergistic effect when two natural compounds are combined. In the current study, the synergistic effect was especially observed at concentrations higher than 6% and 0.3% of HE and TA, respectively. The antibacterial effects of haskap berry extracts have been associated with hydroxycinnamic acids (chlorogenic acid), flavonols (rutin and quercetin-3-glucoside), and organic acids (citric acid, malic acid, quinic acid, and oxalic acid), which are high-level bioactive constituents in their structures. The antibacterial activity mechanism of the phenolic compounds can be attributed to the fact that these alter the structure and function of the cytoplasmic membrane or inactivate cellular enzymes. Many studies have suggested that phenolic compounds primarily target the cytoplasmic membrane and disrupt cell integrity through the accumulation of hydrophobic phenolic groups (hydroxyl groups) in the lipid bilayer [38]. Similarly, several studies have demonstrated that tannic acid has antimicrobial activity against food-borne pathogens. A recent study reported that pomegranate peel polyphenols, which are very rich in hydrolyzable tannins, have a significant antibacterial effect on *C. sakazakii* [39]. Tannic acid has been shown to different antimicrobial mechanisms of action, including iron chelation, inhibition of cell wall synthesis, and disruption of the cell membrane. The antibacterial activity of tannins is mainly attributed to the presence of galloyl group in their structure [40,41]. 

The surface response analysis indicated that the interaction of HE concentration*temperature was another significant effect (*p* = 0.0043) for *C. sakazakii* reduction. It was observed that the treatments in which HE concentrations were higher than 6% and temperatures were above 45 °C caused a geometric increase in *C. sakazakii* inactivation (Figure 3B). In addition, a similar drastic increase in the inhibition of *C. sakazakii* was also observed with the interaction of TA concentration*temperature (Figure 3D), which was an insignificant term (*p* = 0.2186 > 0.05). The obtained results revealed that increasing the HE concentration and the temperature together is much more effective than the TA concentration and time variables in the inactivation of *C. sakazakii*. The drastic increase could be attributed to the synergistic effect, which is exposed with the combination of the antimicrobial agent by mild heat treatment. This phenomenon could be explained by the fact that the heat application caused damage to the bacterial membrane and the phospholipid bilayer more fluid, providing more contact area for the penetration and diffusion of antimicrobial agent [42]. In addition, the enhanced antimicrobial effect at a higher temperature might be due to the increase in the solubility of bioactive compounds. In our previous study [20], we reported that *D*-values obtained at 55 °C for HE (0.87 ± 0.01 min), TA (1.88 ± 0.03 min), and HE + TA (0.48 ± 0.02 min) were significantly lower (*p* < 0.05) than in unsupplemented physiological saline (15.60 ± 0.81 min). Similar results were obtained by Shi et al. [43], who reported that mild heating synergistically enhanced the bactericidal effect of citral against *C. sakazakii*. The antimicrobial effect of citral was more pronounced and rapid at elevated temperatures (50 and 55 °C) compared to a lower temperature (45 °C) in reconstituted infant formula. Jang and Rhee [44] observed a greater reduction in the population of *Cronobacter* spp. at higher temperatures and concentrations of caprylic acid. 

### 3.3. Optimal Conditions for Maximum Reduction in C. sakazakii

Optimal conditions for *C. sakazakii* inactivation were determined using the ’numerical optimization’ application of the Design-Expert program (7.0.0). The program suggested that there could be 25 different possible optimaç conditions that maximize the inhibition of *C. sakazakii*. Ten possible conditions are given in Table 5. The Food and Agriculture Organization (FAO) and World Health Organization (WHO) [45,46] recommended using water at temperatures above 70 °C when preparing powdered infant formula to achieve a 4 logarithmic reduction in the *Cronobacter* spp. counts in infant formulas. However, these temperature values can cause the deterioration of heat-sensitive components (such as protein, vitamins) in infant formula. The basic principle in all food processes is the selection of a temperature and time at which the nutritional elements are preserved at the maximum level while the processing conditions ensure microbiological safety. Based on this principle, it can be concluded that the second solution (9.16% HE concentration, 0.50% TA concentration, 43.84 °C temperature, and 1.05 min) was the best treatment as regards maximizing the reduction at the lowest temperature. However, high concentrations of phenolic extracts, such as 9.16% of haskap extract and 0.5% tannic acid, were needed. On the other hand, we observed that the treatment (eighth solution) which consisted of 3.72% HE concentration, 0.10% TA concentration, 52.59 °C temperature, and 4.94 min, provided a 7.55 logarithmic reduction. An increase of approximately 10 °C in temperature caused a 3-fold and 5-fold decrease in HA concentration TA concentration, respectively, while treatment time extended 5-fold. Therefore, factors such as the preservation of nutritional value, the acceptance of the sensory properties, and economic implications must be given full consideration when selecting the most appropriate treatment. 

## 4. Conclusions

The individual and synergistic effects of HE concentration, TA concentration, temperature, and time on the inhibition of *C. sakazakii* were studied. The results clearly showed that the inhibition of C. sakazakii was considerably influenced by HE concentration, TA concentration, and temperature, while the impact of time was statistically insignificant. Among the variables, temperature was the most important variable, and the response surface plots revealed a synergistic effect of HE concentration*TA concentration and HE concentration*temperature. The different optimal conditions for maximum inhibition of *C. sakazakii* were obtained. The findings revealed that there were two distinct combinations in which the inhibition of *C. sakazakii* was maximized. The first one was high concentration of phenolic extract–low temperature of treatment, while the second was high temperature of treatment–low concentration of phenolic extract. Hence, the pros and cons for mild heat treatments under different conditions should be considered in selecting the optimal conditions by food manufacturers. The primary limitation of this study is that the TA concentrations used were higher than the level allowed by Food and Drug Administration (FDA). Another shortcoming is that this study was not conducted in PIF. Therefore, further studies considering the range of TA levels approved by the FDA are needed to examine the potential risks (e.g., toxicity, interactions with other food ingredients, sensory properties) associated with using haskap extract and TA in infant formula matrices.

## Figures and Tables

**Table 1:** Four-factor–five-level experimental ranges in surface response method central composite design.

		Coded and Actual Values				
Independent Variables	Unit	−2	−1	0	+1	+2
Haskap extract (HE) concentration (*X_1_*)	%	2	4	6	8	10
Tannic acid (TA) concentration (*X_2_*)	%	0.1	0.2	0.3	0.4	0.5
Temperature (*X_3_*)	°C	35	40	45	50	55
Time (*X_4_*)	min	1	2	3	4	5

HE: Haskap extract, TA: Tannic acid.

**Table 2:** Four-factor–five-level central composite design used for RSM and the actual and predicted inhibition values obtained under different conditions.

	Factor 1 (*X_1_*)	Factor 2 (*X_2_*)	Factor 3 (*X_3_*)	Factor 4 (*X_4_*)	Reduction (log cfu/mL) *		
Standard Order	HE Con. (%)	TA Conc. (%)	Temperature (ºC)	Time (min)	Actual	Predicted-1	Predicted-2
1	4.00	0.20	40.00	2.00	0.31	0.14	0.30
2	8.00	0.20	40.00	2.00	0.97	0.51	0.85
3	4.00	0.40	40.00	2.00	0.83	1.40	0.71
4	8.00	0.40	40.00	2.00	2.92	2.89	2.38
5	4.00	0.20	50.00	2.00	2.90	2.83	2.94
6	8.00	0.20	50.00	2.00	4.98	4.90	5.19
7	4.00	0.40	50.00	2.00	3.85	3.46	3.35
8	8.00	0.40	50.00	2.00	7.04	6.65	6.73
9	4.00	0.20	40.00	4.00	0.41	0.60	0.68
10	8.00	0.20	40.00	4.00	1.20	1.34	1.24
11	4.00	0.40	40.00	4.00	0.98	0.80	1.09
12	8.00	0.40	40.00	4.00	2.80	2.66	2.77
13	4.00	0.20	50.00	4.00	4.05	3.83	3.33
14	8.00	0.20	50.00	4.00	7.04	6.27	5.58
15	4.00	0.40	50.00	4.00	3.14	3.40	3.74
16	8.00	0.40	50.00	4.00	7.04	6.96	7.11
17	2.00	0.30	45.00	3.00	1.12	0.90	0.98
18	10.00	0.30	45.00	3.00	4.15	4.83	4.91
19	6.00	0.10	45.00	3.00	0.95	1.44	1.04
20	6.00	0.50	45.00	3.00	3.41	3.38	2.98
21	6.00	0.30	35.00	3.00	0.89	0.70	0.78
22	6.00	0.30	55.00	3.00	7.04	7.69	7.77
23	6.00	0.30	45.00	1.00	2.02	2.31	2.39
24	6.00	0.30	45.00	5.00	2.91	3.08	3.16
25	6.00	0.30	45.00	3.00	1.68	1.77	2.01
26	6.00	0.30	45.00	3.00	1.85	1.77	2.01
27	6.00	0.30	45.00	3.00	1.54	1.77	2.01
28	6.00	0.30	45.00	3.00	2.01	1.77	2.01

* The reductions are expressed as mean values (n = 3). Predicted-1values were calculated from the equation of the quadratic model, Predicted-2 values were calculated from the equation of the reduced quadratic model.

**Table 3:** Fit summary report for the adequacy of the models tested.

***Sequential model sum of squares***						
Source	Sum of square	df	Mean square	*F* value	*p* value Prob > *F*	
Mean	228.73	1	228.73			
Linear	102.97	4	25.74	31.98	<0.0001	
2FI	6.08	6	1.01	1.39	0.2760	
Quadratic	9.29	4	2.32	9.60	0.0008	Suggested *
Cubic	2.39	8	0.30	1.98	0.2334	Aliased
Residual	0.75	5	0.15			
Total	350.21	28	12.51			
***Lack of fit tests***						
Source	Sum of square	df	Mean square	*F* value	*P* value Prob > *F*	
Linear	18.39	20	0.92	22.06	0.0132	
2FI	12.30	14	0.88	21.09	0.0143	
Quadratic	3.02	10	0.30	7.24	0.0650	Suggested *
Cubic	0.63	2	0.31	7.53	0.0677	Aliased
Pure error	0.12	3	0.042			
***Model Summary Statistics***						
Source	Std. Dev.	*R^2^*	*R^2^_adjusted_*	*R^2^_predicted_*	PRESS	
Linear	0.90	0.8476	0.8211	0.7803	26.68	
2FI	0.86	0.8977	0.8375	0.7631	28.78	
Quadratic	0.49	0.9741	0.9463	0.8551	17.60	Suggested *
Cubic	0.39	0.9938	0.9665	0.2543	90.59	Aliased

* It shows the “suggested” model by the Design-Expert software 7.00.

**Table 4:** Regression coefficients and analysis of variance of the quadratic and reduced quadratic model.

Model Term	Quadratic Model				Reduced Quadratic Model		
	k	*F* value	*p* Value		k	*F* value	*p* Value
**Intercept**	47.87				44.95		
***X_1_***	−2.80	95.86	<0.0001 ^a^		−2.54	73.25	<0.0001 ^a^
***X_2_***	9.1	23.44	0.0003 ^a^		−3.54	17.92	0.0005 ^a^
***X_3_***	−2.07	302.94	<0.0001 ^a^		−1.94	231.50	<0.0001 ^a^
***X_4_***	−1.88	3.71	0.0761		−0.95	2.84	0.1093
***X_1_X_2_***	1.40	5.18	0.0404 ^b^		1.40	3.96	0.0620
***X_1_X_3_***	0.04	11.95	0.0043 ^a^		0.04	9.13	0.0073 ^a^
***X_1_X_4_***	0.04	0.56	0.4658				
***X_2_X_3_***	−0.32	1.67	0.2186				
***X_2_X_4_***	−2.64	4.61	0.0512				
***X_3_X_4_***	0.03	1.18	0.2968				
***X_1_^2^***	0.07	7.42	0.0174 ^b^		0.06		0.0501
***X_2_^2^***	15.96	2.53	0.1359				
***X_3_^2^***	0.03	36.44	<0.0001 ^a^		0.02		<0.0001 ^a^
***X_4_^2^***	0.23	5.29	0.0387 ^b^		0.19		0.1030
**Model**		34.97	<0.0001			40.67	<0.0001
***R* ^2^**	0.9741				0.9531		
***R*** **^2^ *_adjusted_***	0.9463				0.9297		
***R*** **^2^ *_predicted_***	0.8551				0.8622		
**CV**	17.20				19.68		
**PRESS**	17.60				16.74		
**Adequate precision**	20.975				22.228		
**Lack-of-fit**	0.0650				0.0484		

Lack of fit is insignificant at *p* > 0.05, ^a^: Significant at *p* < 0.01, ^b^: Significant at *p* < 0.05, *X*_1_: HE concentration, *X*_2_: TA concentration, *X*_3_: Temperature, *X*_4_: Time.

**Table 5:** Possible combinations for maximizing the inhibition efficiency of *C. sakazakii*.

Solution	HE Conc. (%)	TA Conc. (%)	Temperature (°C)	Time (min)	Reduction (log cfu/mL)
1	9.77	0.34	49.60	1.05	8.01
2	9.16	0.50	43.84	1.05	7.32
3	9.59	0.49	49.98	2.09	9.86
4	9.88	0.29	47.17	4.82	7.41
5	9.64	0.46	47.25	4.95	8.49
6	7.95	0.25	53.00	2.53	7.55
7	5.58	0.40	54.50	2.29	7.17
8	3.72	0.10	52.59	4.94	7.55
9	7.86	0.49	50.07	4.91	8.06
10	8.15	0.27	52.25	3.83	8.08

## Data Availability

The original contributions presented in the study are included in the article, further inquiries can be directed to the corresponding author.
